# Elegant Science

**DOI:** 10.1128/mBio.00043-18

**Published:** 2018-02-20

**Authors:** Arturo Casadevall, Ferric C. Fang

**Affiliations:** a Department of Molecular Microbiology and Immunology, Johns Hopkins Bloomberg School of Public Health, Baltimore, Maryland, USA; b Departments of Laboratory Medicine and Microbiology, University of Washington School of Medicine, Seattle, Washington, USA

**Keywords:** elegant, reproducibility, rigor

## Abstract

Elegance is a prized quality in science that is associated with simplicity and explanatory power. This essay considers the qualities that make a scientific model, experiment, method, or theory “elegant,” with a focus on the life sciences. We propose a definition of elegance that includes clarity, cleverness, correctness, explanatory power, parsimony, and beauty. The pursuit of elegance can improve the quality of science, but elegance must be pursued with caution, as the truth is sometimes inelegant.

**Elegance should be left to shoemakers and tailors.**

—Ludwig Boltzmann

## EDITORIAL

Among the qualities most highly valued by scientists is “elegance.” Here we consider the meaning of elegance as part of our ongoing exploration of contemporary science, which has previously included descriptive, mechanistic, important, reproducible, and reductionistic science (1–5). We humbly acknowledge that many who have come before us have found scientific elegance difficult to define. Interest in scientific elegance is not limited to scientists; the subject has been covered in the *New Yorker* (6) and *Atlantic Monthly* (7) magazines. In science, “elegance” and its cousin “beauty” have often been used in the context of physics. According to a 2002 *New York Times* article, the 10 most beautiful scientific experiments of all time were all in the field of physics (8). However, we suggest that elegance can also apply to biology.

The word “elegant” entered the English language in the 1400s, a French word (*élégant*) that was derived from the Latin *elegantem* (9). The *Merriam-Webster Dictionary* defines “elegant” as “tasteful richness of design or ornamentation, dignified gracefulness or restrained beauty of style,” and in the specific context of science, as exhibiting “precision, neatness, and simplicity” (10).

Glynn has argued that the essence of scientific elegance is simplicity and explanatory power, while at the same time noting that its appreciation requires a historical context (11). A recent article celebrating the discovery of the C_60_ molecule buckminsterfullerene noted that an elegant theory or model must explain a phenomenon “clearly, directly and economically” (12). The *New Yorker* article adds that elegance in science requires “simplicity plus capaciousness” in explanatory power (6). Nathan and Brancaccio have gone further, arguing that elegance is “an intrinsic feature of successful scientific practice and observation, a benchmark that demarcates between good experiments and bad ones” (13). This formulation seems to imply that elegance could apply to all good science and that high quality is an intrinsic characteristic of scientific elegance.

The question of whether elegance is merely desirable or essential for good science is particularly relevant to current concerns about rigor and reproducibility in biomedical research (14). If elegance in science is just an attractive attribute, then elegance is not a necessary goal but simply something to be admired when it happens. However, if elegance is a requisite feature of good science, then the characteristics defining elegance deserve the same attention given to scientific rigor. We will unpack these possibilities with examples provided from the biological sciences.

**FIG 1:**
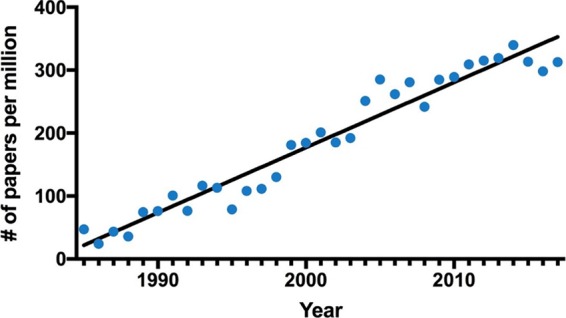
Journal articles in PubMed containing the keyword “elegant” as a function of publication year. The number of papers has been corrected for the number of total journal articles in each year.

A search of the PubMed database for the word “elegant” yields the titles and abstracts of more than 4,000 publications. Browsing through those publications reveals that the word “elegant” is used to describe models, experiments, methods, or theories. Furthermore, the appearance of the word “elegant” in PubMed publications is increasing almost linearly over time. In the mid-1990s, fewer than 100 articles used the word “elegant” in titles and abstracts, whereas in recent years the number of articles using this word now routinely exceeds 300 per year (Fig. 1). Hence, biomedical scientists are increasingly using the word “elegant” in the context of their work.

## EXAMPLES OF ELEGANT SCIENCE

The structure of DNA is often referred to as an elegant model. In 1953, Watson and Crick reported that the structure of DNA is a double helix (15). Pauling had preceded Watson and Crick in proposing a DNA structure based on a triple helix (16). However, the Watson-Crick Model was simpler, accounted for Chargaff’s rules, and immediately suggested a mechanism for replication. In contrast, none of these criteria was met by Pauling’s three-stranded structure. Hence, the double helix was more elegant than the triple-helical model because of its simplicity and greater explanatory power. Subsequent experimental work established the correctness of Watson and Crick’s model. In this example, we note the qualities of simplicity, correctness, and explanatory power that help to define elegance.

The Watson-Crick model made the testable prediction that DNA replication would be semiconservative, providing the basis for the Meselson-Stahl experiment. In 1958, Meselson and Stahl isotopically labeled DNA bases and separated the products by ultracentrifugation to show that each new strand of DNA is built upon a previously existing strand (17). This was called the “most beautiful experiment in biology” by John Cairns (18). The experiment reinforced the Watson-Crick model and ushered in an era of experimentation that continues uninterrupted to this day (19). Again, the elegance of the Meselson-Stahl experiment is derived from its conceptual simplicity and broad explanatory power. Even so, it is noteworthy that the conclusions of the Meselson-Stahl experiment were not immediately accepted due to alternative explanations that were raised at the time (19).

Elegance also applies to the method of PCR developed by Kary Mullis to amplify DNA from minute quantities of single-stranded template (20, 21). PCR-related technologies have had a tremendous impact on numerous fields, including biomedical research, diagnostics, anthropology, and criminology, to name just a few. We have previously argued that the development of PCR can be considered revolutionary science (22). Although the fundamental idea of amplifying DNA by denaturing a DNA target and amplifying a segment with synthetic primers and polymerase was published a decade earlier (23), Mullis had the clever and transformative insight of using a heat-stable polymerase from a thermophilic microbe, which made the method simpler and faster. The elegance of PCR allowed the development of convenient and affordable automated platforms that could perform DNA amplification in a wide range of settings.

The most elegant theory in the biological sciences is unquestionably Darwin’s theory of evolution by natural selection, which was all the more remarkable for being proposed before genes or DNA were known. As with PCR, some of the basic principles underlying natural selection were proposed well before Darwin published *The Origin of Species* (24); new species had been suggested to arise from existing species, and Lamarck had hypothesized that speciation arose in response to environmental demands. Darwin’s crucial insights were that differential fitness of individuals within a population could lead to differences in their survival and reproduction and that the ancestral relationships between species were reflected in the relatedness of their essential characteristics. This theory had the essential virtues of elegance: simplicity, clarity, and explanatory power. In addition, Darwin’s theory had the virtue of being clever. An obvious idea could be simple, clear, and correct with great explanatory power, without necessarily being considered elegant. To be truly elegant, an idea, once proposed, should cause others to exclaim, as T. H. Huxley did, “How extremely stupid not to have thought of that!”

## SIMPLICITY AND ELEGANCE

The theme of simplicity runs deep in elegant science, and definitions of elegance invariably contain such words as “simplicity,” “neatness,” and “economy.” However, it is not immediately obvious that simplicity should be considered necessary or sufficient for scientific elegance. Elegance does not connote simplicity in fashion, as it is hard to imagine a bikini being viewed as elegant. The association of simplicity with elegance in science may relate to the principle of parsimony also known as Occam’s razor, which states that the simplest explanation is the most likely to be correct. This in turn implies that for science to be elegant, it must also be correct. What Occam, a 14th century theologian, actually wrote was “Non sunt multiplicanda entia sine necessitate,” which can be translated as “Entities must not be multiplied beyond necessity.” This principle is not without risk. As Francis Crick observed, “Occam’s razor … can be a very dangerous implement in biology. It is … very rash to use simplicity and elegance as a guide in biological research” (25). He also said that “God is a hacker, not an engineer,” implying that evolution works without foresight and in doing so may solve problems in rather inelegant ways. An elegant theory can actually impede research progress if it delays the appreciation of conflicting observations due to confirmation bias (26).

Nevertheless, we suggest that parsimony is preferable to simplicity, as simplicity in science is relative. For example, when some physicists expressed frustration over the complexity of Einstein’s theories of relativity in comparison to Newton’s formulas, he responded that “It can scarcely be denied that the supreme goal of all theory is to make the irreducible basic elements as simple and as few as possible,” but he cautioned that this must be achieved “without having to surrender the adequate representation of a single datum of experience” (27). This in turn led to the aphorism that “Everything should be made as simple as possible, *but no simpler*.” Hence, there are limitations on the relationship between simplicity and elegance in science.

## A DEFINITION OF SCIENTIFIC ELEGANCE

**FIG 2:**
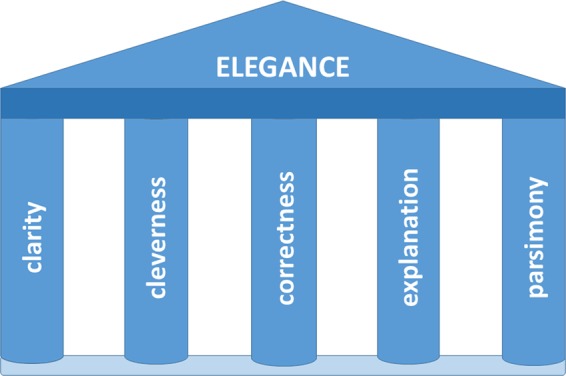
The concept of elegance in science rests on the five pillars of clarity, cleverness, correctness, parsimony, and explanation.

From the foregoing discussion, we propose that to be considered elegant, science should meet the five criteria of being clear, clever, correct, explanatory, and parsimonious (Fig. 2). This concept of scientific elegance parallels our Pentateuch for improving rigor in science (14) in its 5-fold symmetry. The symmetry serves as a reminder that beauty is an important quality in the philosophy of science. Perhaps our definition of elegance also requires a measure of esthetics—is the science beautiful? For example, imagine the complete dissection of a signaling pathway involving dozens of interacting components. Would this be considered elegant science? According to our proposed definition, such a pathway could be considered an example of elegant science, but the beauty of its construction may only be apparent to the cognoscenti. They might argue that such a system can be regarded as beautiful if one considers the challenge of communicating information to the cell rapidly, faithfully, and efficiently, in order to allow an appropriate homeostatic response. Our proposed definition can only provide boundary conditions that are necessary but not sufficient for scientific elegance. To be elegant, a scientific discovery or theory must also meet an esthetic criterion in the mind of the observer. In essence, the discernment of elegant science is analogous to the appreciation of art, which means that the quality of elegance in science is ultimately a human judgment.

## STRIVING FOR ELEGANCE IN SCIENTIFIC RESEARCH

Although scientific rigor (14) is necessary but not sufficient for elegant science, it should be possible to make scientific work more elegant by considering the elements of elegance in research design. One may certainly strive for clarity, correctness, parsimony, cleverness, and explanation when designing experiments, perhaps leaving the more subjective quality of beauty to posterity. Emphasis on clarity and correctness can help to improve the reproducibility of science. However, here one must recall Crick’s admonition that nature sometimes works in inelegant ways; an elegant theory may be seductive and can prevent a researcher from recognizing a less attractive truth. Achieving parsimony requires the generation and consideration of alternative theories, models, or methods that can enhance scientific work by opening a researcher’s mind to other possibilities. Finally, asking whether a proposed experiment will definitively answer a question and explain the problem at hand can improve experimental design. Hence, the components of our definition of elegance (Fig. 2) are mutually self-supporting and represent important elements of good scientific work. The quest for elegance can make science better even when elegance is not achieved. Hence, this quest should be a goal of all scientists.
